# Use of Recombinant Antigens for Sensitive Serodiagnosis of American Tegumentary Leishmaniasis Caused by Different Leishmania Species

**DOI:** 10.1128/JCM.01904-16

**Published:** 2017-01-25

**Authors:** Camila Massae Sato, Maria Carmen Arroyo Sanchez, Beatriz Julieta Celeste, Malcolm S. Duthie, Jeffrey Guderian, Steven G. Reed, Maria Edileuza Felinto de Brito, Marliane Batista Campos, Helia Valeria de Souza Encarnação, Jorge Guerra, Tirza Gabrielle Ramos de Mesquita, Suzana Kanawati Pinheiro, Rajendranath Ramasawmy, Fernando Tobias Silveira, Marina de Assis Souza, Hiro Goto

**Affiliations:** a Instituto de Medicina Tropical de São Paulo, Universidade de São Paulo, São Paulo, Brazil; b Infectious Disease Research Institute, Seattle, Washington, USA; c Department of Immunology, Aggeu Magalhães Research Center-Oswaldo Cruz Foundation (FIOCRUZ), Recife, Pernambuco, Brazil; d Instituto Evandro Chagas, Belém, Pará, Brazil; e Fundação de Medicina Tropical Dr. Heitor Vieira Dourado, Manaus, Amazonas, Brazil; f Universidade do Estado do Amazonas, Amazonas, Brazil; g Universidade Nilton Lins, Manaus, Amazonas, Brazil; h Universidade Federal do Pará/Núcleo de Medicina Tropical, UFPA/NMT, Pará, Brazil; i Faculdade de Medicina da Universidade de São Paulo, São Paulo, Brazil; UNC Health Care System

**Keywords:** ELISA, diagnostics, immunoserology, recombinant antigen, tegumentary leishmaniasis

## Abstract

American tegumentary leishmaniasis (ATL) (also known as cutaneous leishmaniasis [CL]) is caused by various species of protozoa of the genus Leishmania. The diagnosis is achieved on a clinical, epidemiological, and pathological basis, supported by positive parasitological exams and demonstration of leishmanin delayed-type hypersensitivity. Serological assays are not routinely used in the diagnosis because many are considered to have low sensitivity and the particular Leishmania species causing the disease can lead to variable performance. In the present study, we generated recombinant versions of two highly conserved Leishmania proteins, Leishmania (Viannia) braziliensis-derived Lb8E and Lb6H, and evaluated both in enzyme-linked immunosorbent assays (ELISA). Recombinant Lb6H (rLb6H) had better performance and reacted with 100.0% of the ATL and 89.4% of the VL samples. These reactions with rLb6H were highly specific (98.5%) when compared against those for samples from healthy control individuals. We then assessed rLb6H against sera from ATL patients infected with different species of Leishmania prevalent in Brazil [Leishmania (Leishmania) amazonensis, *L*. (Viannia) braziliensis, and *L*. (*V*.) guyanensis] and samples from patients with other infectious diseases. In analyses of 500 sera, ELISA using rLb6H detected all 219 ATL samples (sensitivity of 100.0%) with an overall specificity of 93.9% (considering healthy individuals and other infectious diseases patients). Only a minority of samples from Chagas disease patients possessed antibodies against rLb6H, and all of these responses were low (with a highest reactivity index of 2.2). Taken together, our data support further evaluation of rLb6H and the potential for its routine use in the serological diagnosis of ATL.

## INTRODUCTION

Leishmaniases are the variety of diseases caused by protozoa of the genus Leishmania. Numerous Leishmania species are distributed worldwide, causing a wide range of disease manifestations (1–3). The clinical forms of the disease depend predominantly on the infecting species but are also influenced by the genetic background and immune response of the host. The infection may be asymptomatic or present across a spectrum of clinical manifestations, ranging from localized, disseminated, diffuse or recidiva cutaneous, or mucosal forms, or with involvement of organs such as liver and spleen in cases of visceral leishmaniasis (VL) (3). More than 350 million people, in 88 countries, are at risk of Leishmania infection, and 12 million people are estimated to be infected. The annual incidence is 1.5 million cases for cutaneous leishmaniasis (CL) and 500,000 for VL (4).

American tegumentary leishmaniasis (ATL) (also known as cutaneous leishmaniasis) is characterized by chronic and often latent infection. In some cases, after the appearance of an initial cutaneous lesion, multiple cutaneous lesions (disseminated leishmaniasis) and/or mucosal lesions (mucosal leishmaniasis) can arise as the parasites disseminate through blood and the lymphatic system. The clinical manifestations of ulcerated and nonulcerated lesions during ATL can be similar to those of diseases such as leprosy, paracoccidioidomycosis, syphilis, and cutaneous tuberculosis, among others (5). Laboratory testing is therefore desirable to confirm the diagnosis of ATL. While direct parasite detection is considered confirmatory for the diagnosis of leishmaniasis, direct detection methods have demonstrated low sensitivity that only rises to 88% when they are combined with labor-intensive immunohistochemistry (5). Thus, other indirect immunoassays, such as delayed-type hypersensitivity to Leishmania antigens (leishmanin test) and serological anti-Leishmania antibody tests, are sometimes used (6, 7).

The most commonly used serological assays for the diagnosis of leishmaniasis are indirect immunofluorescence assays, enzyme-linked immunosorbent assays (ELISAs), and Western blots. Initial studies with a large number of samples from ATL/CL patients from northern and northeastern Brazil reported low sensitivities of both an indirect immunofluorescence assay (27.7%) and an ELISA (66.9%) (8). Higher sensitivities were observed with mucosal leishmaniasis (ML), at 56.7% and 93.3%, respectively (9). While the performance of these tests seems to be improving, the sensitivities have been highly variable (75 to 96%) (10).

A sensitive serological assay for the diagnosis of ATL could guide the proper management and treatment of patients. From the perspective of product development and reproducibility, it would be desirable to have recombinant antigens as alternatives to crude antigens that require parasite growth. For VL, assays using recombinant Leishmania antigens are already integrated into the diagnostic routine, and immunochromatographic rapid tests are available to be used at the point of care (11). For diagnosis of ATL/CL, however, there is no consensus on the use of serological assays and on the preferred antigens.

We previously generated promising results with ATL and VL using Leishmania (Leishmania) infantum recombinant Hsp83 (rHsp83) antigen (12), although in that study the ATL samples had no characterization of causative Leishmania species. In the present study, we aimed to determine the capacity of recombinant Leishmania antigens for the serological diagnosis of ATL caused by different species of Leishmania. Leishmania braziliensis-derived recombinant Lb6H (rLb6H) and Lb8E (rLb8E) antigens were generated and used in antibody-detection ELISA evaluating samples from the northern, northeastern, and southeastern areas of Brazil where different Leishmania species prevail. Our data, obtained with 219 patient samples (see Table 1 for the demographic data for patients), indicate 100.0% sensitivity for the confirmation of ATL and 93.9% specificity compared against data for samples from healthy individuals and other infectious diseases patients. Our results raise the possibility of using ELISA with rLb6H (ELISA-rLb6H) in the routine diagnosis for ATL/CL.

**TABLE 1:** Demographic data for ATL patients

Parasite^*a*^	Sex^*b*^		Age (yr)		Disease duration (mo)		Clinical presentation (no.)		
	M	F	Median	Min–max^*c*^	Median	Min–max	Cutaneous	Mucosal	Unidentified
L. amazonensis (*n* = 6)	6	0	34	9–44	96	1.47–300	6	0	0
L. braziliensis (*n* = 115)	59	40	33	13–78	3	0.03–672	65	34	15
L. guyanensis (*n* = 96)	71	14	Not known	Not known	Not known	Not known	96	0	0

a *n*, number of samples.

b M, male; F, female.

c Min–max, minimum to maximum.

## RESULTS

### Conservation of potential serodiagnostic proteins across Leishmania species.

We previously reported high sensitivity and specificity for the serodiagnosis of leishmaniasis using *L*. (*L*.) infantum rHsp83 (12). While this protein was found to be unstable (degraded and yielding inconsistent results over time), the data indicated the potential of using conserved Leishmania proteins in antibody detection assays to assist in the diagnosis of ATL/CL. We therefore evaluated the amino acid sequences of two selected *L*. (Viannia) braziliensis proteins, rLb8E and rLb6H, across available Leishmania sequences. Each of the proteins exhibited >90% identity at the amino acid level with homologs in L. infantum, the causative agent of VL in Brazil, and L. major, a causative agent of CL (Table 2). Identity was slightly reduced in the related kinetoplastid protozoan parasite that is also endemic in Brazil, Trypanosoma cruzi, the causative agent of Chagas disease. Homology data thereby indicate the conservation of Lb8E and Lb6H across Leishmania species and suggest their potential as targets of the infection-induced antibody response.

**TABLE 2:** Amino acid conservation across protozoan parasites

Parasite	Results for 6H:				Results for 8E:			
	No. of aa^*a*^	No. of matched aa	No. of mismatched aa	% identity	No. of aa	No. of matched aa	No. of mismatched aa	% identity
L. braziliensis	657				152			
L. infantum	653	606	51	92	152	140	12	92
L. major	654	608	49	93	152	140	12	92
T. cruzi	657	560	97	85	147	120	32	79

a aa, amino acid.

### Detection of antigen-specific antibodies in sera from leishmaniases patients.

**FIG 1:**
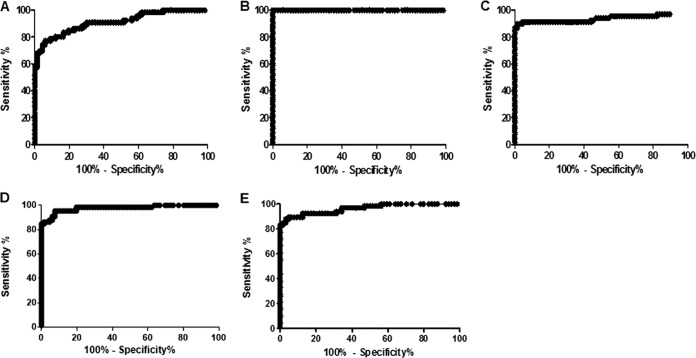
Receiver operating characteristic (ROC) curves to determine ELISA performance. In the top panels, antigen-specific antibody ELISAs were developed with samples from ATL patients (*n* = 68) and healthy subjects (*n* = 68) and are contrasted: (A) ELISA-rLb8E considered a cutoff 36.5, (B) ELISA-rLb6H considered a cutoff 16.0, and (C) ELISA-L. major-like considered a cutoff 10.0. In the bottom panels, antigen-specific antibody ELISAs were developed with samples from visceral leishmaniasis patients (*n* = 66) and healthy subjects (*n* = 66): (D) ELISA-rLb8E considered a cutoff 39.1 and (E) ELISA-rLb6H considered a cutoff 6.1.

In an initial testing round, we contrasted the ability of rLb8E and rLb6H to detect antibodies in sera from leishmaniasis patients against control samples from healthy individuals. Antigen-specific IgG was detected by ELISA and measured in 68 samples from ATL patients (a subset of group I) and 68 samples from healthy individuals (group III), and receiver operating characteristic (ROC) curves were generated (Fig. 1 A, B, and C). With use of the cutoff values determined by ROC curves as providing the best performance characteristics for each antigen, anti-rLb8E IgG was detected in 83.3% of the ATL cases with a specificity of 83.3% (Table 3). Anti-rLb6H IgG was detected in all (100.0%) of the ATL/CL cases with a specificity of 98.5%. This sensitivity was actually improved over that obtained with crude L. major-like antigens, which detected antibodies in 91.2% of the ATL/CL cases at a reduced specificity of 95.6%.

**TABLE 3:** Diagnostic performance of antigens as determined by ROC curves

Presentation^*a*^	Antigen	Cutoff	% sensitivity (95% CI^*b*^)	% specificity (95% CI)	% accuracy
ATL	rLB8E	36.5	83.3 (72.1–91.4)	83.3 (72.1–91.4)	83.3
	rLb6H	16.0	100.0 (94.7–100.0)	98.5 (92.1–100.0)	99.2
	L. major-like	10.0	91.2 (81.8–96.7)	95.6 (87.6–99.1)	91.1
VL	rLB8E	39.1	95.4 (87.3–99.0)	92.4 (83.2–97.5)	93.1
	rLb6H	6.1	89.4 (79.4–95.6)	95.3 (86.9–99.0)	92.3

a Samples used in the calculation of the cutoff versus control samples from healthy individuals. ATL, American tegumentary leishmaniasis; VL, visceral leishmaniasis.

b 95% CI, 95% probability confidence interval.

We similarly evaluated the ability of rLb8E and rLb6H to detect antibodies in sera from VL patients (Fig. 1D and E). Antibodies against rLb8E were found in 95.4% and antibodies against rLb6H in 89.4% of the VL patient sera tested (Table 3). These responses were detected at specificities of 92.4% and 95.3% for rLb8E and rLb6H, respectively. Taken together, these data indicated that rLb6H was an excellent candidate for ATL diagnosis that warranted further investigation.

### Reactivity of rLb6H with sera from ATL cases caused by various Leishmania species.

**FIG 2:**
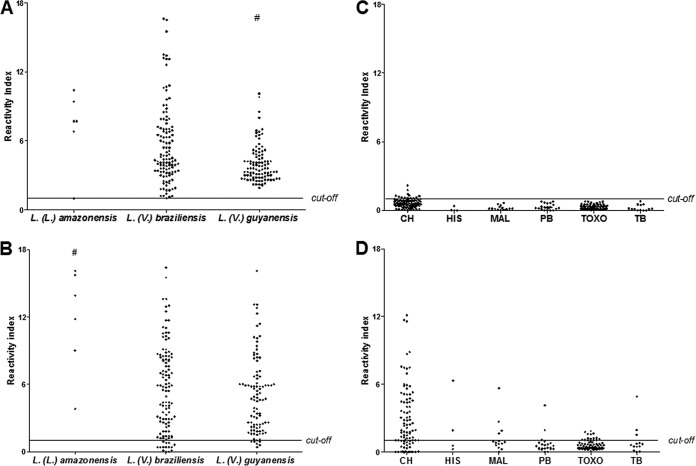
Antigen-specific responses among patients with American tegumentary leishmaniasis (ATL) (A, B) or other infectious diseases (C, D). Antigen-specific antibodies were developed for rLb6H (A, C) and L. major-like proteins (B, D). Serum samples were from ATL patients (group I) and individuals diagnosed with other infectious diseases (group II: Chagas disease [CH], *n* = 91; histoplasmosis [HIS], *n* = 4; malaria [MAL], *n* = 14; paracoccidioidomycosis [PB], *n* = 22); toxoplasmosis [TOXO], *n* = 69; and tuberculosis [TB] *n* = 13). The horizontal line in each plot represents the cutoff at a reactivity index of 1. Kruskal-Wallis one-way analysis of variance on ranks: *P* = 0.0003 (A) and *P* = 0.0088 (B); Dunn's multiple comparison test: *P* < 0.05 (A, B).

Having demonstrated that antibodies against rLb6H were present in the sera of clinically diagnosed ATL patients, we further investigated reactivity by assessing responses in ATL patients for whom the infecting Leishmania species was defined (group I, Table 3). Anti-Lb6H antibodies were readily detected in all ATL cases (95% confidence interval [CI], 98.3 to 100.0) caused by infection with either *L*. (*L*.) amazonensis, *L*. (*V*.). braziliensis, or *L*. (*V*.) guyanensis (Fig. 2). Samples from 2 patients infected with *L*. (*V*.) shawi were strongly reactive, presenting high reactivity index (RI) values (RI = 13.1 and 9.2) (data not shown in Fig. 2). Interestingly, the magnitude of responses varied, depending on the infecting species, with patients infected with *L*. (*V*.) guyanensis (median RI = 3.6) having weaker responses than patients infected with *L*. (*L*.) amazonensis (median RI = 7.7) and *L*. (*V*.) braziliensis (median RI = 4.5). For L. major-like antigens, stronger responses were detected with patients infected with *L*. (*L*.) amazonensis (median RI = 12.9) than with *L*. (*V*.) braziliensis (median RI = 5.5) or *L*. (*V*.) guyanensis (median RI = 4.7). When responses against rLb6H were contrasted against those detected by L. major-like antigens, it was again apparent that the recombinant antigen was more reactive with sera from ATL patients than the crude antigens (Fig. 2A and B). Anti-L. major-like antibodies were detected in 90.9% (95% CI, 86.3 to 94.0) of the ATL patient sera tested. Thus, this expanded analysis further supports the potential of using rLB6H for the serodiagnosis of ATL/CL.

### rLb6H has minimal reactivity with serum samples from other infectious diseases.

Considering the very high sensitivity of the ELISA-rLb6H in detecting ATL cases, we were concerned that this might have arisen at the expense of specificity. We therefore evaluated specificity under more stringent conditions, assessing the ELISA-rLb6H with 213 serum samples from patients with other infectious diseases. Among 91 samples from Chagas disease patients, 74 (81.3%) were below the threshold and 17 (18.7%) were low positive (the reactivity index ranged from 1.0 to 2.2) (Fig. 2C and D). These results were in stark contrast with those generated with L. major-like antigens, for which a majority (75.8%) of Chagas disease samples reacted. In addition, at least two samples from each of the other diseases assessed possessed antibodies that could bind L. major-like antigens (Table 4). Thus, without, or with minimal, interference from other potentially confounding pathogens, the ELISA-rLb6H appears suitable for the serodiagnosis of ATL/CL.

**TABLE 4:** Antigen-specific antibodies in sera of patients with potentially confounding infectious diseases

Disease	No. of samples	ELISA-rLb6H		ELISA-L. major-like	
		No. reactive (%)	95% CI^*a*^	No. reactive (%)	95% CI
Chagas disease	91	17 (18.7)	12.0–27.9	69 (75.8)	66.1–83.5
Histoplasmosis	4	0 (0.0)	0.0–49.0	2 (50.0)	15.0–85.0
Malaria	14	0 (0.0)	0.0–21.1	6 (42.9)	21.4–67.4
Paracoccidioidomycosis	22	0 (0.0)	0.0–14.9	4 (18.2)	7.3–38.5
Toxoplasmosis	69	0 (0.0)	0.0–5.3	15 (21.7)	13.6–32.8
Tuberculosis	13	0 (0.0)	0.0–22.8	3 (23.1)	8.2–50.3
Total	213	17 (8.0)	5.0–12.4	99 (46.7)	39.9–53.2

a 95% CI, 95% probability confidence interval.

## DISCUSSION

Diagnosis of ATL/CL can be difficult due to its varied clinical manifestations that have similarities with fungal and mycobacterial infections, among other diseases (13). To date, laboratory tests such as parasitological and histopathological exams have exhibited low sensitivity and the diagnostic utility of the leishmanin (delayed-type hypersensitivity) test is limited because it indicates not only current infection but also prior infection. The general belief has been that serological assays for ATL/CL would be limited by low sensitivity, and they have not therefore been considered for use in routine diagnosis. Serological assays are, however, used in some institutions although many work with antigens produced in-house from Leishmania promastigotes grown in culture, such that a universal, standardized antigen is lacking (14–16).

In recent decades, an increasing number of recombinant proteins have been described as candidates for the diagnosis of leishmaniasis (17–21). In general, however, the sensitivity of these antigens has been low and has not warranted further development into serologic tests that can be widely used. We previously obtained high sensitivities and specificities for ELISA using Leishmania (*L*.) infantum rHsp83 against ATL and VL samples (12). The rHsp83 antigen is unstable and degrades, however, and this has precluded its further development into a standardized diagnostic method. rHsp83 did provide proof of concept for the potential of antibody detection assays to assist the diagnosis of ATL and indicated the importance of identifying new and highly conserved proteins among different species of Leishmania to provide antigens for detection of antibodies in sera of ATL patients. We therefore evaluated the reactivity of Leishmania (*V*.) braziliensis-derived rLb8E and rLb6H antigens in sera from leishmaniasis patients in this study. While both antigens performed very well (high sensitivity and specificity for Leishmania infection), initial testing indicated that rLb6H had a greater capacity then rLb8E to react with serum antibodies of ATL patients. Reactivity with VL samples does not necessarily constitute a major problem for the diagnosis and management of patients since the clinical presentations of VL and ATL/CL are typically distinct. Exceptions may occur in cases of HIV-Leishmania coinfection that may have unusual manifestations (22), but a positive antibody response in this situation may prompt the use of direct detection methods for verification of the infecting Leishmania species and appropriate management of the patient.

In the present study, the performance of rLb6H was indicated to be excellent, yielding 100.0% sensitivity and 98.5% specificity (versus healthy controls) for the detection of antibodies in sera from ATL patients. Furthermore, our cohort consisted of samples of patients from different Leishmania-endemic regions of Brazil for whom ATL was caused by the particular species of Leishmania prevalent to the region. Previous reports have suggested that variations in assay sensitivity may occur due to the infecting Leishmania species. When *L*. (*L*.) amazonensis antigen preparations were used for assaying, sensitivities were observed to be slightly lower with samples from patients infected by *L*. (*V*.) guyanensis than from patients infected by *L*. (*V*.) braziliensis: 79.6% and 71.7% by indirect immunofluorescence assay and of 98.2% and 85.0% by ELISA, respectively. Furthermore, for both tests, the titers with samples from *L*. (*V*.) guyanensis-infected patients were significantly lower than those with samples from *L*. (*V*.) braziliensis-infected patients (23). It is noteworthy that the reactivity against rLb6H observed in our study tended to be higher with samples from patients infected with *L*. (*L*.) amazonensis, followed by those infected with *L*. (*L*.) braziliensis and then those infected with *L*. (*V*.) guyanensis. Thus, our data suggest that the amount of circulating antibody may be dependent upon the Leishmania species involved. There are data suggesting variations of anti-Leishmania antibody levels dependent on clinical presentations of the disease (24), but in the present study, the available data did not allow us to address this aspect, which will be further explored. Although our data also suggested that reactivity levels varied considerably among groups of patients when the duration of disease was longer, no correlation was detected (Spearman correlation, *r* = 0.052, *P* = 0.7192).

Rigorous evaluation of sera from other diseases against rLb6H indicated that the recombinant protein provided much greater specificity for ATL than that achieved with Leishmania major-like total antigen. The only apparent concern arose when sera of patients with Chagas disease were evaluated, with 18.7% of the Chagas disease samples exhibiting low-level responses (the highest reactivity index measured was 2.2). While this is likely a result of cross-reactivity due to the high degree of homology of the 6H antigen between *L*. (*V*.) braziliensis and T. cruzi, we cannot formally exclude the possibility that the Chagas disease patients were also latently infected with Leishmania. Any suspicion of coinfection could be further investigated using Chagas disease-specific tests to verify T. cruzi infection or parasitological examinations to exclude/confirm Leishmania infection. Regardless, the results obtained with the rLb6H antigen are very promising, supporting further evaluation and potential for routine use in the serological diagnosis of ATL.

## MATERIALS AND METHODS

### Patients and serum samples.

Serum samples from 500 Brazilian adults of both sexes were classified into three groups. Group I consisted of 219 samples of ATL patients from regions where different Leishmania species are present: the northern city of Manaus, Amazon state, and various cities of Para state; the northeastern city of Recife, Pernambuco state; and the southeastern city of São Paulo, Sao Paulo state (Table 1). Leishmaniasis was diagnosed by demonstration of a positive leishmanin test (25), histopathology of skin lesions (26, 27), and/or direct identification of Leishmania infection by either parasitological exams or PCR (27). The species identified as causative agents of ATL were *L*. (*L*.) amazonensis, *L*. (*V*.) braziliensis, *L*. (*V*.) guyanensis, and *L*. (*V*.) shawi. Various methods were used to identify the infecting species: monoclonal antibodies (28) (samples from Para state), PCR-restriction fragment length polymorphism (RFLP) of Hsp70 (29, 30) (samples from Manaus), and schizodemas (31) (samples from Recife). To assess specificity and potential cross-reactivity, group II consisted of 213 samples from patients with other diseases: Chagas disease (*n* = 91), histoplasmosis (*n* = 4), malaria (*n* = 14), paracoccidioidomycosis (*n* = 22), toxoplasmosis (*n* = 69), and tuberculosis (*n* = 13). These latter samples were obtained from other studies, all approved by the ethics committees of the institutions involved. Twenty Chagas disease samples, in particular, were from Montes Claros, Minas Gerais state. Group III consisted of 68 individuals considered healthy by their own assessment and/or clinical exam. The study was approved by the Conselho Nacional de Ética em Pesquisa (National Council of Ethics on Research) and the Ethics Committee of Faculdade de Medicina, Universidade de São Paulo (protocol 490.392).

### Antigens.

The Lb6H and Lb8E antigens were originally identified during screening of a *L*. (*V*.) braziliensis genomic expression library with serum from a ML patient (32). Oligonucleotide PCR primers were designed to contain a 5′ NdeI site, followed by residues encoding an amino-terminal six-histidine tag, and a 3′ EcoRI site following the residues encoding the termination codon. The PCR-amplified product was digested with NdeI and EcoRI and ligated into pET 17b and subsequently transformed into Escherichia coli BL-21 host cells for expression. Recombinant Lb6H was purified from the insoluble inclusion bodies, and recombinant Lb8E was purified from the soluble fraction by affinity chromatography using a nickle-nitrilotriacetic acid (Ni-NTA) agarose column. The purified protein fractions were analyzed by sodium dodecyl sulfate-polyacrylamide gel electrophoresis (SDS-PAGE) and quantified using the BCA protein assay (Pierce, Rockford, IL).

The total alkaline extract of L. major-like (MHOM/BR/71/49) was produced according to the method of Celeste et al. (33).

### Recombinant Leishmania antigen-specific and L. major-like total antigen-specific antibody detection ELISAs.

Polystyrene plates were coated with 50 μl/well of either 1 μg/ml rLb6H and rLb8E antigens or 5 μg/ml L. major-like total antigen in 0.06 M carbonate-bicarbonate buffer [pH 9.6]. Plates were incubated overnight at 4°C in a humidified chamber and then washed three times with 0.01 M phosphate-buffered saline (pH 7.2) containing Tween 20 (polyoxyethylene-sorbitan monolaurate; Sigma-Aldrich, St. Louis, MO, USA) (PBS-T). Wells were blocked by adding 125 μl/well of PBS-T containing 5% skimmed milk (PBS-T-L 5%) and incubating at 37°C for 2 h in a humidified chamber before washing three times with PBS-T. Then, samples diluted either 1/100 (for the recombinant antigen ELISA) or 1/160 (for the total L. major-like ELISA) in PBS-T-L 2% were added in duplicate wells (50 μl/well) and incubated at 37°C in a humidified chamber for 30 min before washing five times with PBS-T. Then 50 μl/well of goat anti-human IgG peroxidase conjugate (Calbiochem, La Jolla, CA, USA) diluted 1/20,000 in PBS-T-L 2% was added and incubated at 37°C in a moist chamber for 30 min before the plates were washed five times with PBS-T. Finally, 50 μl/well of tetramethylbenzidine-H_2_O_2_ (TMB) single solution (Novex; Life Technologies, Carlsbad, CA, USA) was added, and the reaction was allowed to progress for 7 min at room temperature under dark conditions before being stopped by the addition of 25 μl/well of 2 N H_2_SO_4_. The spectrophotometric reading of each well was performed at 450 nm using a Multiskan GO instrument (Thermo Scientific, Finland).

### Statistical analysis.

The sensitivity, specificity, and confidence intervals (95% CI) of each assay were calculated after construction of a receiver operating characteristic (ROC) curve. The cutoff value to discriminate negative and positive samples was determined by ROC curves as providing the best performance following analyses of 68 samples from ATL patients and 68 samples from healthy individuals. The ELISA results were expressed as the optical density (OD) of the test sample divided by the OD of the positive control in each plate and multiplied by 100, called percentage point positivity (pp) of the positive control (34). For each sample, the reactivity index (RI) was calculated by dividing the pp value per cutoff. Statistical analysis was performed using GraphPad Prism 5 (GraphPad Software Inc., San Diego, CA, USA) and SigmaStat 3.5 analysis system software (Systat Software, Richmond, CA, USA). The Kruskal-Wallis one-way analysis of variance on ranks followed by Dunn's multiple comparison test, when appropriate, was used to compare medians. The correlation between reactivity levels and the duration of disease were assessed by Spearman's rank correlation test. Differences were considered statistically significant when *P* values were <0.05.
